# Interplay between Immune Cell Infiltration and Tumor Histological Subtype: A Case of Adrenocortical Cancer

**DOI:** 10.3390/cancers14215303

**Published:** 2022-10-28

**Authors:** Apollinariya V. Bogolyubova, Nano V. Pachuashvili, Arina V. Tkachuk, Natalia G. Mokrysheva, Liliya S. Urusova

**Affiliations:** 1 National Research Center for Hematology, 125167 Moscow, Russia; 2 Institute of Clinical Morphology and Digital Pathology, I.M. Sechenov First Moscow State Medical University, 119991 Moscow, Russia; 3 Endocrinology Research Centre, 117036 Moscow, Russia

**Keywords:** tumor microenvironment, tumor-infiltrated immune cells, histological variant, adrenocortical cancer, oncocytic adrenocortical cancer

## Abstract

**Simple Summary:**

The interplay between histological subtype and tumor microenvironment, especially the presence of tumor-infiltrating immune cells (TIIC), in adrenocortical cancer remains unclear. Here, we perform a comparative analysis of TIIC composition in conventional and oncocytic histological variants of adrenocortical cancer (ACC) using cases from The Cancer Genome Atlas database and the Endocrinology Research Centre collection (Moscow, Russia) and provide strong evidence that oncocytic ACC is characterized not only by improved survival but also by intensive immune infiltration compared to conventional ACC. Thus, these two histological variants should be independently analyzed to determine the potential role of TIIC in survival and tumor progression.

**Abstract:**

The analysis of the tumor microenvironment, especially tumor-infiltrated immune cells, is essential for predicting tumor prognosis, clinical outcomes, and therapy strategies. Adrenocortical cancer is a rare nonimmunogenic malignancy in which the importance of the presence of immune cells is not well understood. In our study, we made the first attempt to understand the interplay between the histology of adrenocortical cancer and its immune landscape using cases from The Cancer Genome Atlas database and the Endocrinology Research Centre collection (Moscow, Russia). We showed that the oncocytic variant of adrenocortical cancer is characterized by intensive immune infiltration and better survival, and it is crucial to analyze the effect of immune infiltration independently for each histological variant.

## 1. Introduction

Adrenocortical cancer (ACC) is a rare malignant tumor of the adrenal cortex, usually characterized by a late detection, aggressive clinical course, and poor outcome [1,2]. Due to the difficulties of diagnosing ACC, the tumor is usually detected at the stage of metastasis, limiting the possibilities of surgical intervention, and suggesting a poor prognosis [3,4]. Mitotane, an oral chemotherapeutic agent, is the primary drug for the treatment of ACC, but, unfortunately, it is often poorly tolerated; therefore, its use is limited [5]. The strategy of ACC therapy has not changed in recent years; currently used cytotoxic chemotherapy includes drugs that demonstrate a limited efficacy and high toxicity [6,7,8].

**Figure 1:**
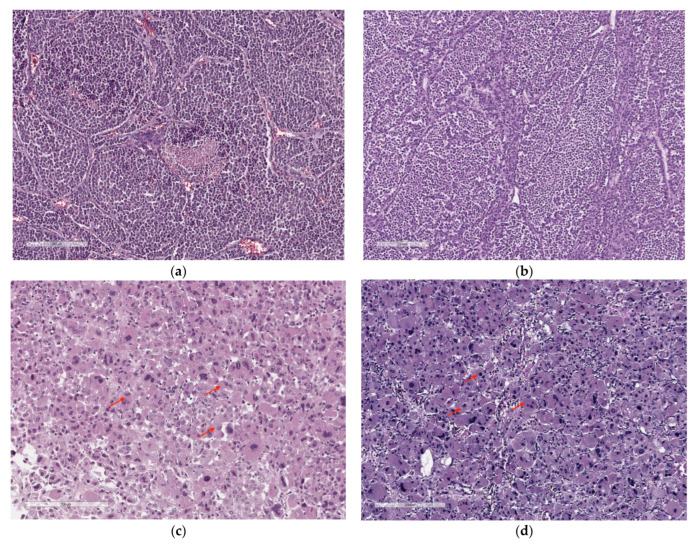
Hematoxylin and eosin staining of samples of conventional (**a**,**b**) and oncocytic (**c**,**d**) histological variants of ACC. Red arrows indicate the representative oncocytic cytology characterized by an abandoned granular eosinophilic cytoplasm.

There are several histological variants of ACC, including conventional [9] (Figure 1a,b), oncocytic [10] (Figure 1c,d), myxoid [11,12], and sarcomatoid variants [1,13,14,15]. The first two are predominant. The classic criteria of Weiss and colleagues described in 1984 and modified in 1989 continue to be used for the classification of conventional adrenal cortical neoplasms in adults [16,17]. The new WHO classification also expands the use of other multiparameter diagnostic algorithms, these include the reticulin algorithm (for conventional, oncocytic, and myxoid histological variants) [18,19], the Lin–Weiss–Bisceglia system (for the oncocytic histological variant) [10], and the Helsinki scoring system (for conventional, oncocytic, and myxoid histological variants) [20,21].

An oncocytic variant consists of about 18% of all ACC cases and is characterized by a specific cytological peculiarity of cancer cells (presence of granular eosinophilic cytoplasm, diffuse growth pattern, and high-grade nuclei) [22]. Oncocytes are up to two times larger than acinar cells. The presence of eosinophilic cytoplasm is due to the accumulation of defective mitochondria, which in some cases can occupy most of the cytoplasm and sometimes displace other cytoplasmic organelles [23]. The tumor cells of oncocytic ACC form solid, trabecular, or glandular structures [24,25]. An immunohistochemical analysis with antimitochondrial antibodies (AMA) can distinguish between oncocytic and conventional ACC [26], but in most cases, it can be conducted through a routine microscopic examination of high-quality hematoxylin and eosin slides [27]. In 2021, a study of 75 patients was published, in which they characterized the above-described histological variants of the tumor with a description of growth patterns [28]. The study also presented a minimum panel of immunohistochemical markers required for the diagnosis of ACC, which included: SF1, Melan A, and Inhibin A.

Tumor microenvironment (TME) composition is critical for the development of immunotherapy treatment strategies of cancer. One of the components of TME is immune cells, the presence of which is usually associated with an improved prognosis for cancer patients [29,30]. Most recently, Georgantzoglou et al. published a review to study the characteristics of the adrenocortical cancer microenvironment [31]. However, the composition of the tumor-infiltrating immune cells (TIIC) in ACC, especially in different histological variants, is not thoroughly understood.

Currently, there are many computational algorithms for evaluating the composition of the TIIC based on RNA-Seq data, such as CIBERSORT [32], CIBERSORTx [33], ConsensusTME [34], MCP-counter [35], TIMER [36], and others. These algorithms can potentially allow us to search for immune cell composition in samples from databases, such as The Cancer Genome Atlas (TCGA, https://portal.gdc.cancer.gov/, accessed on 20 October 2022). This is specifically useful for rare cancer types, including ACC, for which it might be impossible to achieve the representative cohort of patient’s samples in one institution.

There are some publications on the effect of TIIC on the survival and prognosis of ACC based on the TCGA data. For example, Tian X et al. reviewed 79 ACC cases from the TCGA database and reported that a higher amount of tumor-infiltrating mast cells positively correlated with the outcome of ACC patients [37]. In addition, a recent study from Baechle et al. demonstrated that mast cell infiltration was strongly associated with a favorable prognosis in ACC patients and marked key anti- and protumor genes, in which expression was significant for tumor growth [38].

In our study, we focused on the interplay between histological variants and TIIC in ACC based on the data from the TCGA dataset and data obtained from our sample collection from the Endocrinology Research Centre (Moscow, Russia). We demonstrated that the oncocytic histological variant of ACC demonstrated higher parameters of TIIC compared to conventional ACC and this is associated with improved survival in patients with oncocytic ACC.

## 2. Materials and Methods

### 2.1. Patients and Samples

The study involved two ACC cohorts: patients from the TCGA database and patients treated at the Endocrinology Research Centre (ERC).

Data from 78 patients with available RNA-Seq were taken from The Cancer Genome Atlas (TCGA) database. Furthermore, differential diagnosis was performed by three independent pathologists based on high-resolution images of tumor sections available in the Cancer Digital Slide Archive (https://cancer.digitalslidearchive.org/, accessed on 20 October 2022) [39]. For some cases, only low-quality frozen tissue sections were available, which could not be used to accurately determine the histological variant of ACC unambiguously. Therefore, such cases were not included in further analysis. Finally, a total of 54 patients were included in the TCGA cohort (Table S1). During the revision, the presence of 28 cases (52%) of the conventional and 21 cases (39%) of the oncocytic variant of ACC was shown. Three cases (5%) of myxoid and two cases (4%) of sarcomatoid ACC were also described. Notably, the sarcomatoid ACC cases coincided with those identified by Zheng et al. based on genetic analysis [40].

Another cohort of ACC represented paraffin-embedded tumor tissue samples with detailed clinical and pathological description from patients treated at the ERC between 2010 and 2020. The samples included 21 cases, which were identified as conventional (10 cases, 48%), oncocytic (9 cases, 43%), and myxoid (2 cases, 9%) variants of ACC (see Table S2 for detailed clinical information).

Each of the 21 patients underwent histological diagnostics and a series of immunohistochemical stains for the markers of the main immune cell subsets (see below for further details). The study was approved by the Research Ethics Committee of the Endocrinology Research Centre (ethical approval protocol #10, 26 May 2021).

### 2.2. Analysis of the TIIC Composition Using RNA-Seq Data

The analysis of the representation of TIIC in the tumor tissue of samples from the TCGA cohort was carried out using four different methods.

Firstly, data were taken from Thorsson et al. [29] as it is represented in Supplementary Table S1. Briefly, we used the CIBERSORT [32] algorithm applied to a re-quantification using Kallisto and the Gencode GTF TCGA data. Secondly, we applied the CIBERSORTx algorithm [33] to TCGA data without re-quantification using the following parameters: LM22 signature matrix file, B-mode batch correction, disabled quantile normalization, relative run mode, set to 100 permutations. Thirdly, ConsensusTME [34] analysis was performed by us using the R script from the original paper (https://github.com/cansysbio/ConsensusTME, accessed on 20 October 2022). Finally, we used the data of TIIC representation from Bagaev et al. [41], which are freely available at https://science.bostongene.com/tumor-portrait/ (accessed on 20 October 2022).

### 2.3. Immunohistochemistry and Histological Imaging

Immunohistochemical analysis of tumor tissue sections from the ERC cohort was carried out according to the standard technique with a peroxidase detection system with DAB on an automatic Leica BOND III IHC staining system using Leica reagents and protocols. Antibodies to CD45 (PA0042, Leica), CD3 (PA0553, Leica), CD4 (PA0427, Leica), CD8 (PA0183, Leica), and CD68 (PA0273, Leica) were used. All histological slides were scanned using a Leica Aperio AT2 system at 20× magnification for further analysis.

### 2.4. Immune Cells Counting

The counting of immune cells was carried out in five fields of view with a size of 0.05 mm^2^ (the total area was 0.25 mm^2^) apart for the stroma and parenchyma of the tumor (ERC cohort). The most representative fields of view were selected, and the exact localization was chosen for different markers.

### 2.5. Statistical Analysis

The results were statistically processed using IBM SPSS Statistics 26 (Cox regression) and GraphPad Prism 6 (Kaplan-Meier curves, Spearman’s correlation).

## 3. Results

### 3.1. The Survival of Patients with Conventional and Oncocytic Variants of ACC Significantly Varies

The heterogeneity of ACC is becoming better understood in the modern era: it turns out that the oncocytic variant, which was previously considered to be extremely rare, is actually quite common, and can account for up to 40% of all ACC tumors. Thus, the diagnosis which figures in the TCGA database should be additionally verified by examining high-resolution scanned slides, which are available at the Cancer Digital Slide Archive (https://cancer.digitalslidearchive.org/, accessed on 20 October 2022) [39].

We carried out a detailed verification of the diagnosis of all ACC cases presented in the TCGA database for a further survival analysis of ACC cases separate from each histological variant (see Section 2 and Tables S1 and S2 for further details).

**Figure 2:**
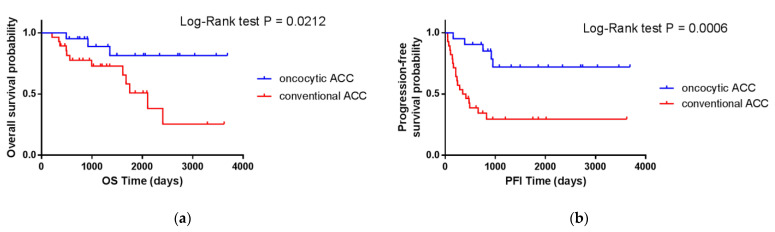
Overall (**a**) and progression-free (**b**) survival of conventional and oncocytic ACC patients from the TCGA cohort.

**Figure 3:**
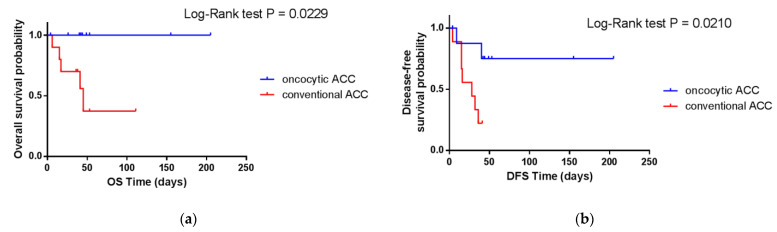
Overall (**a**) and disease-free (**b**) survival of conventional and oncocytic ACC patients from the ERC cohort.

The analysis of the prognosis for patients with conventional and oncocytic ACC revealed significant differences between the two given groups in both cohorts of this study. Figure 2 and Figure 3 shows the Kaplan–Meier curves for overall (OS) and progression-free (PFI) survival for the TCGA cohort and overall (OS) and disease-free (DFS) survival for the ERC cohort, respectively, which clearly demonstrate this difference. The differences in survival between conventional and oncocytic histological variants of ACC were statistically significant (*p*-value < 0.05).

We also observed a correlation between the OS and PFI and the histological variant of ACC in the TCGA cohort. The Spearman’s correlation coefficients were 0.307 (*p*-value = 0.0320) and 0.436 (*p*-value = 0.0017), respectively.

### 3.2. The Intensity of the Immune Infiltration Differs between Histological Variants of ACC

To address the question of the potential differences in the intensity of the tumor immune infiltration between conventional and oncocytic variants of ACC, we performed an immunohistochemical analysis of markers of different immune cell types for samples from the ERC cohort. We analyzed CD45^+^ pan-leukocyte infiltration and CD3^+^, CD4^+^, and CD8^+^ T cell infiltration as well as the presence of CD68+ macrophages (see Table 1 for details).

The number of CD45^+^ immune cells in tumor parenchyma and stroma was relatively low (176 and 225 cells/mm^2^, respectively). However, the number of immune cells from all the analyzed populations in tumor parenchyma was higher in oncocytic compared to conventional ACC cases.

A further assessment of the intensity of the immune infiltration was performed for the TCGA cohort using data of the representation of TIIC obtained from RNA-seq analysis.

**Figure 4:**
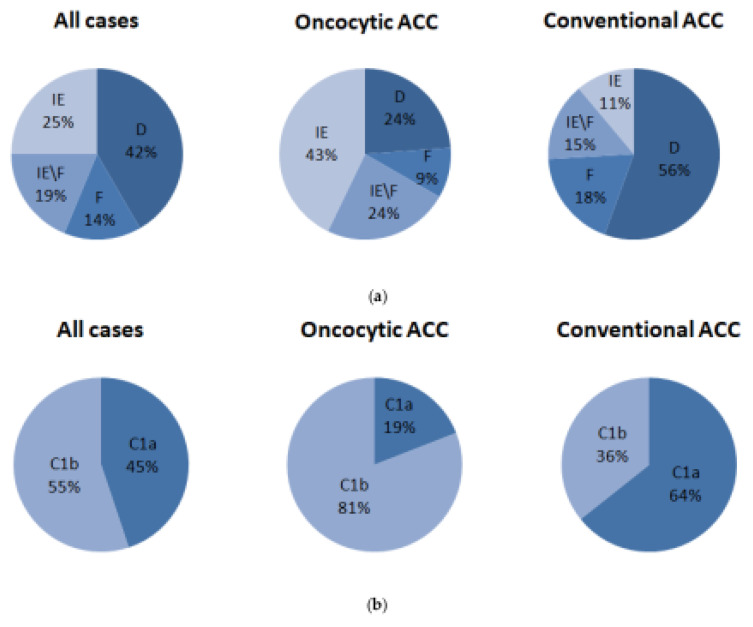
Distribution of the TCGA tumors by different immune clusters: (**a**). According to Bagaev et al. [41]: D—immune depleted, F—fibrotic, IE—immune enriched, IE/F—immune enriched, fibrotic samples; (**b**). According to Zheng et al. [40]: C1a—steroid-high, C1b—steroid-low/immune-high clusters.

According to Bagaev et al. [41], tumors from the TCGA cohort were distinguished for four clusters: (1) immune enriched, fibrotic (IE/F), (2) immune enriched, nonfibrotic (IE), (3) fibrotic (F), and (4) immune depleted (D). It turned out that the largest percentage of ACC tumors (40%) exhibited the D phenotype; the IE and IE/F phenotypes were observed in 24% and 21%, respectively (Figure 4a). Interestingly, the distribution of phenotypes in the conventional and oncocytic variants of ACC differed: in the conventional ACC, cases with immune-depleted phenotypes prevailed, while in the oncocytic ACC, the opposite was true: the prevailing set of tumors (43%) demonstrated the immune-enriched phenotype.

TCGA ACC can be also classified into steroid-low/immune-high (C1b) and steroid-high (C1a) clusters, according to Zheng et al. [40]. We demonstrated that the distribution of these two tumor classes differed between conventional and oncocytic ACC, and the immune-high group was predominant in oncocytic ACC (Figure 4b).

**Figure 5:**
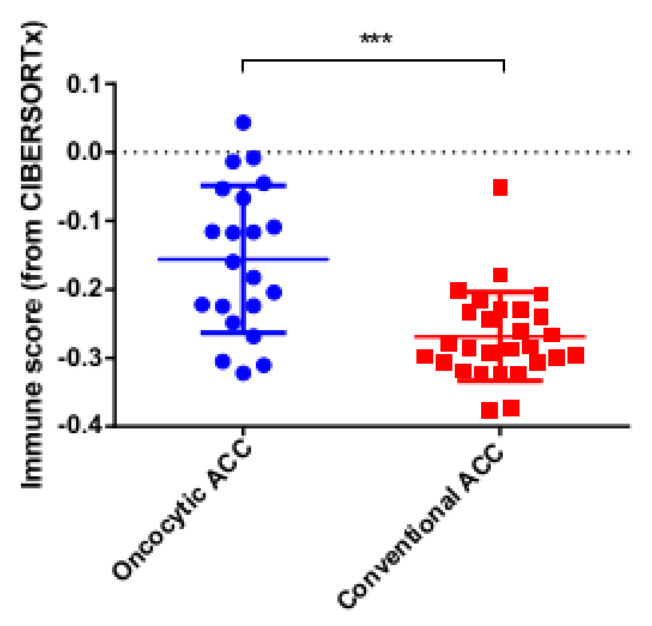
The immune score of TCGA ACC tumor [16] samples based on CIBERSORTx algorithm (*** *p*-value = 0.0002).

For additional analyses, we used the immune score parameter from the CIBERSORTx algorithm [33] to assess the intensity of the immune infiltration. The same result was obtained: the oncocytic variant of ACC was significantly and strongly infiltrated by immune cells, compared to conventional ACC (Figure 5).

The Spearman’s correlation coefficients for indicators such as immune score (CIBERSORTx [33], ConsensusTME [34]), leukocyte fraction [40], and histological tumor type were 0.525 (*p*-value = 0.0001) and 0.496 (*p*-value = 0.0003), respectively. Thus, these parameters are dependent from each other.

## 4. Discussion

The tumor microenvironment (TME) plays a critical role in tumor growth, survival, and prognosis in patients. The TME composition in some cancer types, including melanoma and lung cancer, remains well understood. However, several rare cancers, including ACC, have not yet been well-characterized. For instance, our study of the immune microenvironment in Warthin-like papillary thyroid carcinoma has been published recently [42]. This rare histological variant is determined by the presence of abundant leukocyte infiltration of the tumor stroma. In our work, the subpopulation composition of tumor-infiltrating T-lymphocytes (CD4+, CD8+, CD45RO+) for eight tumor tissue samples was characterized by immunohistochemistry. Nevertheless, the deep characterization of the TIICs presented in the Warthin-like papillary thyroid cancer is not performed yet. For another rare cancer named malignant pleural mesothelioma some data regarding its tumor microenvironment have also been published [43].

There are several papers that attempted to describe the composition of TIIC in ACC. For example, Huang et al. [44] performed an analysis of TIIC in metastatic ACC from the TCGA database based on the CIBERSORT algorithm, showing that several subpopulations of TIIC (such as memory B cells and CD4 memory T cells) may be used as independent predictors of the prognosis for ACC patients. Li et al. [45] demonstrated that a lower immune score estimated by the ESTIMATE algorithm was associated with the poor survival of patients with ACC from the TCGA. Next, Tian X et al. [37] performed the analysis of the positive correlation between the amount of tumor-infiltrating mast cells and the outcome of ACC patients from the TCGA; these observations were confirmed by Baechle et al. [38].

All the above-mentioned studies did not include the contribution of the histological variant in their observed correlations. However, ACC can be divided into several histological variants with different prognoses for patients. In our research, we performed a survival analysis of ACC patients from the TCGA database and the independent ERC cohorts with different histological variants and the intensity of immune infiltration. We provided a strong conclusion that oncocytic ACC is characterized by intensive immune infiltration and better prognosis. It seems that the histology of a given tumor is a stronger prognostic factor than the immune infiltration. Therefore, it is important to independently search for the role of the TIIC in cancer prognosis to each histological variant.

## 5. Conclusions

Most studies of the TIIC based on RNA-seq data consider either the entire TCGA dataset or focus on one of the cancer types. Nevertheless, one should not forget that within each of the tumors, there are several histological subtypes. Notably, they are often distinguished not only due to the cytological characteristics of the tumor cells themselves, but also by the number of TIIC. Accordingly, when considering all tumors of the same localization in one dataset, the relationship between the TME and, for example, the prognosis of patients, may be incorrectly interpreted.

Using simple analysis, we showed the role of histological subtype and immune infiltration intensity on ACC samples in two cohorts. We demonstrated that intensive immune infiltration is common for the oncocytic histological variant of ACC, which is characterized by improved survival rates. Accordingly, for a deeper analysis of the role of various subpopulations of TIIC on tumor progression, cases of conventional and oncocytic ACC must be viewed independently of each other.

## Figures and Tables

**Table 1:** Presence of different immune cell subtypes in tumor parenchyma and stroma of cases from ERC cohort.

		Count (Cells/0.25 mm^2^)		
		All Cases	Conventional ACC	Oncocytic ACC
Parenchyma	CD45+	176 (range 23–560)	153 (range 23–459)	223 (range 27–560)
	CD3+	41 (range 7–144)	34 (range 7–135)	50 (range 11–144)
	CD4+	13 (range 0–81)	8 (range 0–33)	21 (range 2–81)
	CD8+	21 (range 2–93)	17 (range 2–93)	28 (range 5–89)
	CD68+	47 (range 2–207)	27 (range 2–106)	75 (range 2–207)
Stroma	CD45+	225 (range 50–570)	250 (range 116–570)	221 (range 115–317)
	CD3+	125 (range 9–238)	153 (range 69–232)	113 (range 9–238)
	CD4+	47 (range 1–157)	46 (range 1–73)	58 (range 4–157)
	CD8+	54 (range 1–222)	71 (range 23–222)	39 (range 9–80)
	CD68+	58 (range 2–232)	61 (range 2–168)	64 (range 3–232)

## Data Availability

The data presented in this study are available in this article.

## Supplementary Materials

The following are available online at https://www.mdpi.com/article/10.3390/cancers14215303/s1, Table S1: Clinical and histological characteristics of cases from the TCGA cohort included in the study, Table S2: Clinical and histological characteristics of cases from the ERC cohort.
